# 

**Published:** 1848-07-01

**Authors:** 


### To Correspondents.

*Dr. Burnett's* "Insanity tested by Science" will be reviewed in our next number.

*M.D.*, who writes respecting the conduct of the Commissioners in Lunacy in the asylum with which he is associated, is informed that we cannot interfere in these private matters. He has, if aggrieved, his proper legal remedy. Independently of other objections, the personality of the letter would exclude it from our pages.

*Dr. A.*—We know nothing of the society for the protection of persons unjustly confined in asylums on the plea of insanity. We question whether there exists any gross cases of the kind requiring the interposition of any other authority than that legally constituted to take cognizance of this matter.

*Mr. J. S.*—The case to which he refers is, we understand, to be made the subject of judicial investigation. Pending these proceedings, we cannot publish any statement of the case.

*Enquiries.*—Mr. Lutwidge is secretary to the Commissioners in Lunacy. He will give our correspondent the information he requires.

*Justice.*—We believe, according to the strict provisions of the Act for the Regulation of Asylums, there is *no appeal* from the decision of the Commissioners to a higher tribunal.

*Mr. A. Smith* complains of our not openly advocating the modern opinions on mesmerism and phrenology. We purposely avoid identifying our Journal with any party. When the proper time arrives, we shall not be found backward in giving our honest opinions of both these questions.

"*A Physician connected with a Public Asylum*" has not read the article or work with sufficient attention. Dr. Seymour *does not* advocate the administration of sedatives in *all* cases of mental derangement. He maintains that there exists a large class of cases, for the cure of which morphia must be perseveringly exhibited. He has found the sedative plan of treatment of most essential service in cases of melancholia alternating with violence. Dr. Seymour is far from recommending an indiscriminate use of morphia. Of course it requires experience and judgment to select the proper cases for this mode of treatment; and our correspondent must not complain of failures if he finds the morphia of no service when given without reference to the judicious rules which Dr. Seymour has laid down for the guidance of those disposed to give this medicine a fair trial. We have certainly witnessed extraordinary results from the sedative plan of treatment; and great, very great credit is due to Dr. Seymour for bringing the subject so fully before the profession. In our next number we purpose discussing at some length the sedative plan of treatment.

*A. N. D.'s* letter is full of objections. He

"Is nothing, if not critical."

We see no valid reason for changing the name of the Journal. The word "Psychological" is fully comprehended by the learned professions, and that satisfies us.

*Madame de Wahl's* interesting work on the "*Moral, Mental, and Physical Training of Girls at School*" has been received, and will be noticed in the next number of the Journal.

*Scrutator.*—Apply to Miss Cockburn, the matron of the Institution for Idiots, at Highgate. The lady in question will be found very intelligent, and will give our correspondent every information on the subject.

*Dr. J., of Boston,* can obtain copies of the Journal by applying to Wiley and Putnam, the American publishers, in Paternoster-row.

*Surgeon.*—Back numbers of the Journal are to be obtained of the publisher. The first volume will appear in October next, with a full index.

*Mr. K. L.*—The Commissioners in Lunacy, New-street, Spring-gardens. The office he refers to is that of the *Masters in Lunacy,* Lincoln's-inn-fields. They were formerly denominated "Commissioners," but by a new Act of Parliament they became the Masters in Lunacy, taking rank with Masters in Chancery.

*Hanwell.*—The medical treatment of insanity will be fully discussed in our next number.

*Americus.*—We have received no copies of the "American Journal of Insanity;" neither have any reports of American asylums been forwarded to the publisher for the Editor.

*Dr. S.* will hear from us privately.

*X. Y. Z.*—The article will be acceptable. It is desirable that he should take a practical view of the subject.

*A. B., Gibraltar.*—The "Phrenological Journal" has ceased to exist. We know of no magazine having been published in its stead in Scotland.

*Dr. G., Paris.*—The reports will be acceptable. The subject is novel. Its discussion, however, may lead to some practical result.

*Judicial.*—When the trial takes place, a full report will appear in the Journal.

